# Iron need attention: a review on the role of iron in plants

**DOI:** 10.3389/fpls.2026.1876801

**Published:** 2026-09-11

**Authors:** Ruirui Li, Long Ma, Mengyang Li, Xiaoyu Zhu, Yunlong Zhai, Jiahao Liu, Desheng Wang, Lili Yang

**Affiliations:** 1 College of Agriculture, Tarim University, Alar, China; 2 Key Laboratory of Tarim Oasis Agriculture (Tarim University), Ministry of Education, Alar, China

**Keywords:** abiotic stress, antioxidants, iron homeostasis, reactive oxygen species, stress resilience

## Abstract

Plants must continuously adapt to complex dynamic environmental conditions throughout their entire life cycle. Abiotic stresses, such as drought, salinity, heavy metal toxicity, extreme temperatures significantly impair plant growth and cause substantial reductions in crop yield. Iron (Fe), an essential micronutrient, serves as a cofactor for numerous metalloenzymes and a critical component of the mitochondrial and chloroplast electron transport chains. It plays an indispensable role in fundamental physiological processes such as photosynthesis, respiration, antioxidant defense, and nitrogen metabolism. However, abiotic stress frequently disrupts iron homeostasis in plants, resulting in either iron deficiency or toxic accumulation, a dual imbalance that compromises cellular function. Such dysregulation exacerbates oxidative damage via Fenton chemistry and severely inhibits growth and development by impairing photosynthetic efficiency and enzymatic activity. Emerging evidence indicates that iron acts not merely as a nutritional element but also as a key modulator of intracellular signaling, metabolic reprogramming, and ion homeostasis, thereby contributing critically to abiotic stress resilience. This review comprehensively synthesizes current understanding of iron’s regulatory roles in plant responses to major abiotic stresses, with particular emphasis on the crosstalk between iron homeostasis networks and stress activated signaling pathways. We further elucidate how perturbations in iron homeostasis exert bidirectional effects on stress tolerance, both sensitizing and, under certain contexts, potentiating adaptive responses, thereby offering conceptual and mechanistic insights to advance research on the integration of iron nutrition and stress adaptation, and to inform strategies for enhancing crop resilience.

## 1 Introduction

Abiotic stresses (drought, salinity, extreme temperatures, and heavy metal contamination) represent major threats to global food security (Rai et al., 2024).Such adverse environmental conditions severely impair plant growth and development, disrupt physiological metabolism, and substantially reduce both crop yield and quality (Li et al., 2024b). In recent decades, the accelerating pace of climate change and the worsening extent of environmental pollution have markedly intensified the detrimental effects of abiotic stress on agricultural productivity. Collectively, these stresses account for over 50% of global crop yield losses and affect approximately 91% of the world’s arable land (Oyebamiji et al., 2024).

Fe occupies a central regulatory role in the molecular, biochemical, and physiological mechanisms governing plant adaptation to abiotic stress. As an essential micronutrient, Fe is indispensable for core cellular processes, including photosynthesis, mitochondrial and chloroplast respiration, antioxidant defense, and symbiotic nitrogen fixation (Gupta et al., 2024). From chlorophyll biosynthesis to electron transfer in photosynthetic and respiratory electron transport chains, Fe functions as both a structural cofactor and a redox active catalytic center across multiple metabolic pathways (Schmidt et al., 2020). Moreover, Fe modulates the activity of key antioxidant enzyme, such as catalase (CAT), peroxidases, and superoxide dismutase (SOD), thereby enhancing the plant’s capacity to scavenge reactive oxygen species (ROS) and sustain intracellular redox homeostasis under stress conditions (Tavanti et al., 2021). Yet Fe exhibits a well-documented dual functionality: while physiological concentrations are vital for metabolic integrity, both deficiency and excess disrupt cellular ion homeostasis and provoke oxidative damage (Zhang et al., 2022). To maintain iron homeostasis and prevent detrimental consequences, plants have evolved complex transcriptional regulatory networks, members of the basic helix-loop-helix (bHLH) transcription factor family, to tightly control Fe uptake, translocation, assimilation, and storage. These regulatory networks are further intertwined with the perception of other environmental cues (Hanikenne et al., 2021; Vélez-Bermúdez and Schmidt, 2022).

Critically, the interaction between iron homeostasis and abiotic stress is highly stress specific. Under drought, iron accumulation dynamics are altered (Kabir et al., 2025). Rhizosphere bacterial iron metabolism also correlates with drought, and loss of plant phytosiderophore transporters reshapes the microbial community (Pugnaire et al., 2019). Under osmotic stresses like salinity and drought, mineral uptake is impaired, with the transcription factor FIT acting as a hub integrating iron deficiency and ABA-mediated stress responses (Schwarz et al., 2020). Under temperature stress, disrupted iron homeostasis drives root morphological plasticity in wheat via iron-dependent hormonal signaling (Shen et al., 2026). Additionally, drought and salinity induce root cell wall lignification and suberization, reinforcing apoplastic barriers that restrict uncontrolled iron movement and alter apoplastic iron pool dynamics (Liu et al., 2023; Kanwar and Bauer, 2026).

Despite substantial progress in elucidating individual Fe uptake and signaling components, several critical knowledge gaps remain (Saidi et al., 2025). Despite recent progress, our understanding of the spatiotemporal dynamics and molecular crosstalk governing the simultaneous response to Fe deficiency and abiotic stress remains fragmentary. This review addresses these gaps by synthesizing emerging evidence on Fe homeostasis as a central regulatory node within the plant ionome. We examine the evolutionary plasticity of Fe acquisition strategies, encompassing Strategy I (reduction-based), Strategy II (chelation-based), and microbiome-assisted Strategy III, shaped by genotype-soil-microbiome interactions. Furthermore, we dissect long-distance transport mechanisms involving xylem-localized citrate-Fe^3+^ and phloem-localized nicotianamine-Fe^2+^ complexes, alongside subcellular sequestration into ferritin nanocages and vacuoles to balance bioavailability against Fenton-mediated toxicity. We further explore how abiotic stresses perturb this fine-tuned equilibrium causing spatial heterogeneity and oxidative damage, whereas optimal Fe status enhances resilience via coordinated antioxidant responses. thereby offering conceptual and mechanistic insights to advance research on the integration of iron nutrition and stress adaptation, and to inform strategies for enhancing crop resilience. To provide a comprehensive overview of the molecular players involved in these processes, we have summarized the key iron-related genes, their biological functions, and associated regulatory pathways in **Supplementary Table 1**.

## 2 Fe is involved in the physiological processes of plants

### 2.1 Functions in photosynthesis

Fe is indispensable for photosynthetic function, with approximately 80% of cellular Fe localized in chloroplasts (Gratz et al., 2021). Although Fe is not incorporated into the chlorophyll molecule itself, it serves as an essential cofactor for multiple enzymes, such as glutamyl-tRNA reductase, ferro chelatase, and Mg chelatase, that catalyze sequential steps in chlorophyll biosynthesis (Ning et al., 2023). During the light-dependent reactions, iron serves as an essential constituent of Photosystem I (PSI), Photosystem II (PSII), the cytochrome b6f complex, Fe-S clusters, and ferredoxin. It directly governs the efficiency of electron transfer from photoexcited chlorophyll to NADP^+^, as well as the establishment of the transmembrane proton gradient (López-Millán et al., 2016).

Under abiotic stress conditions such as drought, salinity, and high temperature, plants face a severe conflict between impaired iron acquisition and metabolic demand. This manifests as a critical contradiction between the inhibition of root iron uptake capacity and the surging iron requirement in chloroplasts (Kanwar et al., 2021). Drought reduces soil water content, thereby slowing the mass flow and diffusion rates of Fe^2+^ to the root surface. Concurrently, the diminished transpirational pull caused by water deficit hinders the long-distance transport of iron from roots to the shoots. In saline environments, high concentrations of Na^+^ and Cl^-^ not only disrupt membrane potential stability but also exert competitive inhibition against micronutrients, interfering with the normal function of iron transporters and impeding both root iron absorption and its allocation to leaves (Ning et al., 2023). Furthermore, a complex crosstalk exists between osmotic stress signaling and iron deficiency signaling; for instance, the FIT transcription factor may modulate abscisic acid (ABA) responses under stress, dynamically adjusting the expression of iron uptake genes and causing plants to enter an iron-restricted state at the early stages of stress (Schwarz and Bauer, 2020). High-temperature stress drastically increases the demand for iron within chloroplasts, required for repairing damage and maintaining antioxidant defense systems, destabilizing iron-sulfur enzymes and accelerating the turnover of pigment-protein complexes (Suzuki et al., 2012). This “double squeeze” of reduced supply and increased demand renders photosynthetic tissues highly susceptible to functional iron deficiency, even when soil iron levels are sufficient, thereby amplifying the negative impact of stress on photosynthesis. Since the core subunits of PSI (e.g., PsaA, PsaB) and its terminal electron acceptor, ferredoxin, are rich in oxygen-labile [4Fe-4S] clusters, iron deficiency leads to impaired synthesis and assembly of these clusters, resulting in functional damage to PSI and downstream ferredoxin. It also indirectly inhibits the activity of key enzymes in the Calvin cycle, such as Rubisco, by disrupting chloroplast redox homeostasis, ultimately causing a decline in photosynthetic quantum yield and severely limiting carbon assimilation capacity (Zandalinas et al., 2020; Ohnishi et al., 2021). Under high ROS environments induced by drought or heat, improperly assembled PSI complexes are highly prone to photoinhibition and irreversible degradation, causing severe impairment of the electron transport chain at the PSI level (Strzepek and Harrison, 2004). Additionally, iron deficiency inhibits ferro chelatase activity, hindering the conversion of chlorophyll precursors to heme, which subsequently affects the function of the cytochrome b6f complex and weakens the capacity to establish the trans-thylakoid proton gradient required to drive ATP synthesis (Balk and Pilon, 2011; Sági-Kazár et al., 2022). This blockage in electron transport not only reduces the production of NADPH and ATP but also leads to excessive accumulation of excitation energy at reaction centers, triggering intense photo-oxidative damage manifested as rapid leaf chlorosis and the collapse of photosynthetic efficiency (Timperio et al., 2007; Ohnishi et al., 2021).

To cope with stress-exacerbated iron scarcity, plants transcriptionally activate the expression of NAS and transporters to enhance long-distance iron transport in the phloem, prioritizing iron supply to developing young leaves and meristems (Zhang et al., 2019; Rai et al., 2021). At the level of chloroplast homeostasis regulation, plants optimize iron flux into chloroplasts by modulating the activity and abundance of chloroplast inner envelope reductases, particularly FRO7, while simultaneously inducing ferritin expression to chelate free iron and prevent oxidative stress triggered by Fenton reactions (Vigani et al., 2019; Sági-Kazár et al., 2021). In terms of metabolic remodeling, plants downregulate the abundance of highly iron-demanding complexes such as PSI and cytochrome b6f, relatively maintain the PSII ratio, and promote cyclic electron flow (CEF) to sustain necessary ATP synthesis under ferredoxin limitation, thereby meeting the energy costs of stress repair (Chen et al., 2020; Schmidt et al., 2020). Furthermore, by enhancing non-photochemical quenching (NPQ) and the activity of the antioxidant enzyme system, plants effectively dissipate excess excitation energy and scavenge ROS, thereby maximizing the maintenance of thylakoid membrane structural integrity and the functional stability of the photosynthetic electron transport chain under iron-limited conditions (Dos Santos et al., 2021; Shi et al., 2022). These multidimensional, coordinated adaptive mechanisms collectively constitute a critical line of defense for plants to maintain photosynthetic carbon assimilation and survival capabilities under complex adverse conditions.

### 2.2 Respiration and energy metabolism

Fe is an essential cofactor for mitochondrial energy metabolism. Mitochondria must import iron from the cytoplasm to synthesize two major cofactors: Fe-S clusters and heme. These cofactors are then integrated into mitochondrial proteins that support critical functions, including cellular respiration (Jain and Connolly, 2013). Fe-S clusters serve as key prosthetic groups. They ensure electron transfer reactions, activate substrates for catalysis, facilitate cofactor biosynthesis, and stabilize proteins. Mitochondria have a particularly high demand for Fe-S clusters because the activity of respiratory chain complexes I, II, and III relies on their correct assembly and functioning (Przybyla-Toscano et al., 2021). The *de novo* assembly of Fe-S clusters occurs on ISCU scaffold proteins. This process utilizes iron, sulfur, and electron delivery proteins. It requires the coordinated participation of cysteine desulfurase NFS1, ferredoxin, and frataxin (López-López et al., 2022; Pedroletti et al., 2023). The mitochondria specific NFU4 and NFU5 proteins function as Fe-S cluster assembly factors indispensable for protein lipoylation and mitochondrial dehydrogenase function; double nfu4 nfu5 knockout mutants are embryonic lethal, and depletion of NFU4 and NFU5 proteins leads to growth arrest of young seedlings (Przybyla-Toscano et al., 2022). The ISC Fe-S cluster biosynthetic pathway is crucial for iron and sulfur homeostasis in plants. In Arabidopsis thaliana, the mitochondrial cysteine desulfurase (AtNFS1) is indispensable for this assembly process (Armas et al., 2019). Heme biosynthesis likewise centers on the mitochondria, and together with Fe-S clusters provide indispensable metallic prosthetic groups for the oxidative phosphorylation system (Connorton et al., 2017).

Fe-S proteins and heme-containing enzymes (e.g., Complexes I and II, aconitase) constitute an integrated redox network critical for photosynthesis, respiration, and DNA metabolism (Mendoza-Cózatl et al., 2019). Iron and sulfur are pivotal mineral nutrients for these respiratory proteins (Vigani and Briat, 2016). The TCA cycle serves as the metabolic hub, oxidizing acetyl-CoA to generate reducing equivalents (NADH, FADH_2_) that drive the ETC. Iron facilitates continuous electron flow to oxygen via Fe-S centers and heme groups across Complexes I-IV. Under iron deficiency, impaired Fe-S and heme biogenesis disrupts the assembly of TCA enzymes and respiratory complexes, leading to mitochondrial dysfunction and metabolic alterations such as citric acid accumulation (Vigani et al., 2018). The absence of functional iron cofactors collapses the proton motive force and OXPHOS, severely compromising energy-intensive processes like cell division and biosynthesis. Therefore, iron uptake, distribution, and metabolism are tightly regulated at transcriptional and post-transcriptional levels to prevent toxic accumulation while ensuring adequate supply for enzyme cofactors (Connorton et al., 2017).

Plant mitochondria possess unique alternative respiratory pathways, distinct from animal cells, characterized by a branched network that includes non-energy-conserving routes. The alternative oxidase (AOX) pathway is pivotal; it bypasses the cytochrome c pathway to divert electrons directly from ubiquinone to oxygen. While this sacrifices ATP generation efficiency, it is indispensable for maintaining cellular redox homeostasis and metabolic flexibility, particularly under stress. AOX functions to dissipate excess reducing equivalents in the mitochondrial matrix, thereby preventing over reduction of the electron transport chain and mitigating photooxidative stress (Garmash, 2021). Its expression is induced by NO and negatively regulated by the transcription factor MYB29 (Zhang et al., 2017; Gupta et al., 2018). Furthermore, impairments in the respiratory chain, such as those caused by iron deficiency or the knockdown of the mitochondrial iron transporter (MIT), trigger the activation of these alternative pathways to sustain metabolic function (Vigani and Zocchi, 2009; Vigani et al., 2016).

At the systemic adaptation level under iron deficiency, plants adapt to iron deficiency not only through alternative respiration but also by remodeling root metabolic networks to enhance iron acquisition. Given the reactivity and potential toxicity of iron and sulfur, plants have evolved tight regulatory mechanisms for their uptake and assimilation. Evidence suggests a dynamic crosstalk between iron and sulfur networks, mediated by mitochondrial retrograde signaling. Mitochondria-derived reactive ROS and the PAP/SAL1 pathway act as primary triggers, communicating organelle status to the nucleus to coordinate the expression of genes involved in iron and sulfur metabolism (Crawford et al., 2018; Balparda et al., 2020; Khan et al., 2024). Through this integrated regulation of iron-dependent systems and retrograde signaling, plants sustain indispensable bioenergetic capacity and metabolic resilience under abiotic stress.

### 2.3 Antioxidant defense system

**Figure 1:**
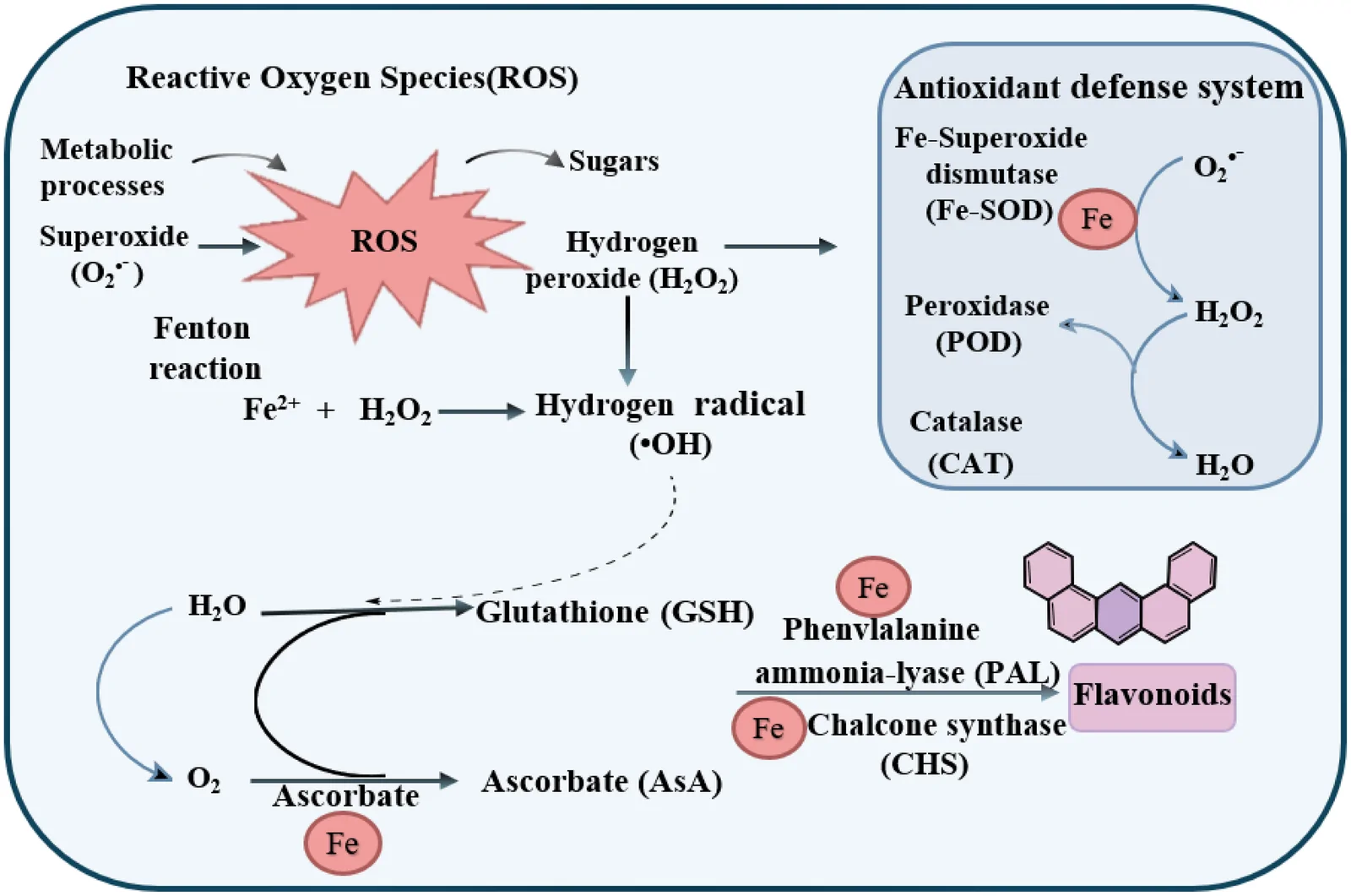
Schematic diagram of reactive oxygen species (ROS) metabolism and the antioxidant defense system in plants. The diagram illustrates the generation of ROS through metabolic processes and the Fenton reaction. It the role of iron as a cofactor in both ROS production and scavenging enzymes, including Fe-Superoxide dismutase (Fe-SOD), Peroxidase (POD), and Catalase (CAT). Additionally, it depicts the Ascorbate-Glutathione (AsA-GSH) cycle and the phenylpropanoid pathway leading to Flavonoids synthesis, emphasizing the involvement of Fe-dependent enzymes such as Phenylalanine ammonia-lyase (PAL) and Chalcone synthase (CHS).

Through evolutionary adaptation, plants have developed a sophisticated, multilayered antioxidant defense system that is essential for scavenging ROS and maintaining intracellular redox homeostasis (Rao et al., 2025). This system underpins plant tolerance, acclimation, and resilience to abiotic stressors (**Figure 1**). Antioxidants are broadly classified into enzymatic and non-enzymatic categories (Song et al., 2020). Enzymatic antioxidants include SOD, POD, and CAT (He et al., 2019). Non-enzymatic antioxidants comprise low molecular weight metabolites such as asredox cycl ingcorbate, glutathione (GSH), tocopherols, and flavonoids (Szarka et al., 2012). Crucially, Fe serves not merely as a structural cofactor but as a central regulatory node integrating both arms of this defense network. Fe is incorporated into the active sites of key antioxidant enzymes (e.g., Fe SOD, heme containing PODs, and catalase), thereby determining their structural integrity and catalytic competence (Miller, 2004). Under abiotic stress, a direct biochemical connection links micronutrient status to cellular redox homeostasis (Tavanti et al., 2021). This integration tightly couples ROS (e.g., superoxide anion and H_2_O_2_) scavenging with iron acquisition and utilization metabolism, thereby preventing oxidative damage accumulation without compromising signaling fidelity. Moreover, Fe availability modulates the transcriptional and post translational regulation of antioxidant enzyme genes and influences the biosynthesis and redox cycling of non-enzymatic antioxidants, particularly GSH and ascorbate (Fourcroy et al., 2004; Genç and Karaman, 2024). Thus, optimal Fe status orchestrates a coordinated upregulation of enzymatic capacity and non-enzymatic buffering, maximizing cellular redox flexibility under stress.

Abiotic stresses induce excessive accumulation of ROS in plant tissues, triggering oxidative damage to lipids, proteins, and nucleic acids (Rodrigues-Corrêa and Fett-Neto, 2019). Fe, when maintained at physiologically optimal levels, enhances antioxidant capacity through multiple coordinated mechanisms (Tewari et al., 2021). Fe serves as an essential cofactor for key enzymatic antioxidants: it is incorporated into the active sites of Fe-SOD and heme containing POD, thereby augmenting their catalytic efficiency in scavenging O_2_^•^-^^ and decomposing H_2_O_2_ (Hwang et al., 2000). This dual action mitigates membrane lipid peroxidation, commonly quantified by malondialdehyde (MDA) accumulation, and preserves cellular integrity under stress. Fe critically regulates the phenylpropanoid pathway, the primary biosynthetic route for flavonoids, by influencing the activity and expression of rate-limiting enzymes, including phenylalanine ammonia-lyase (PAL), cinnamate 4-hydroxylase (C4H), 4-coumarate: CoA ligase (4CL), and chalcone synthase (CHS) (Liu et al., 2021). Under Fe deficiency, PAL and CHS activities are significantly suppressed, resulting in diminished flavonoid accumulation; conversely, adequate Fe supply upregulates both enzyme activities and transcript levels, promoting flavonoid biosynthesis (Caramanico et al., 2017; Liu et al., 2024a). Structurally, flavonoids possess multiple ortho-dihydroxy (catechol) and α-hydroxyketone moieties that confer high affinity chelating capacity for Fe^2+^/Fe^3+^ (Kejík et al., 2021). Flavonoid-Fe complexes serve a dual physiological function: (i) under Fe deficient conditions, they enhance Fe solubility and long distance transport via the xylem, improving Fe acquisition and utilization efficiency; and (ii) under Fe excess, they sequester labile Fe pools, thereby suppressing Fenton chemistry (Fe^2+^ + H_2_O_2_ → Fe^3+^ + ^•^OH + OH^-^) and attenuating hydroxyl radical–mediated oxidative damage (Kejík et al., 2021; Wang et al., 2021). Thus, flavonoids act as redox active Fe buffers, dynamically fine tuning intracellular Fe bioavailability and ROS homeostasis in a concentration dependent manner.

During abiotic stress, enzymatic and non-enzymatic antioxidants operate in a tightly coordinated, interdependent network, constituting the plant’s integrated antioxidant defense system (Hasanuzzaman et al., 2020). Fe modulates this system through both direct biochemical and indirect signaling mechanism (Liang, 2022). This enzymatic regulation reinforces functional synergy between GSH dependent non-enzymatic detoxification and Fe dependent enzymatic antioxidants (e.g., Fe-SOD, catalase), enhancing overall ROS scavenging efficiency. Furthermore, Fe functions as a bona fide signaling molecule: it influences the transcriptional activation or repression of antioxidant-related genes by modulating conserved stress responsive signaling pathways, including Ca^2+^ dependent kinases, mitogen-activated protein kinase (MAPK) cascades, and hormone mediated networks (Mazhar et al., 2023; Yang et al., 2024; Zhao et al., 2024). Under Fe deficiency, plants activate a transcriptionally coordinated response: upregulating expression of Fe acquisition genes (e.g., IRT1, FRO2) while simultaneously inducing antioxidant enzyme genes (e.g., Fe-SOD, GR, APX) to counteract deficiency associated oxidative stress (Walker and Connolly, 2008; Liang, 2022). Third, Fe engages in crosstalk with key endogenous signaling molecules, such as Ca^2+^, ABA, jasmonic acid (JA), and salicylic acid (SA) to fine-tune antioxidant responses. For example, under drought stress, Fe potentiates ABA signaling by enhancing phosphorylation of downstream ABA responsive element binding factors (AREBs) and promoting the expression of ABA inducible antioxidant genes, thereby reinforcing cellular redox homeostasis, and improving drought resilience (Zhang et al., 2020a; Pandey et al., 2024).

### 2.4 The role of iron in plant rhizobia symbiosis

Fe in legume-rhizobia symbiosis extends far beyond its function as a structural cofactor for nitrogenase and leghemoglobin; it serves as a critical hub where nutrient availability intersects with abiotic stress responses. Although an optimal iron concentration (approximately 40 µM in the rhizosphere) is essential for root nodule organogenesis and infection thread progression, environmental stresses such as drought, salinity, and heat significantly disrupt iron homeostasis, thereby compromising symbiotic nitrogen fixation (Zhou et al., 2024; Ren et al., 2025). Recent mechanistic insights reveal that abiotic stress not only induces general physiological decline but also specifically targets iron transport and signaling pathways, creating a bottleneck in the supply of this micronutrient to the symbiotic interface (Li et al., 2024c). Drought stress profoundly alters the dynamics of iron acquisition and distribution in legume roots, directly impacting root nodule development and function. Under water deficit conditions, plants often exhibit reduced iron uptake from the soil due to diminished mass flow and impaired root growth, leading to systemic iron deficiency even when soil iron levels are sufficient (Araki et al., 2022). More critically, drought triggers specific molecular responses that restrict iron delivery to root nodules. For instance, the accumulation of inorganic nitrogen, which frequently occurs under stress conditions that inhibit symbiotic nitrogen fixation, exacerbates iron deficiency in root nodules by suppressing the expression of NRAMP2 transporters. This suppression blocks iron delivery to infected cells, leading to reduced symbiotic nitrogen fixation efficiency despite the presence of functional root nodules (Banasiak et al., 2021). Furthermore, genome resolved metagenomic studies indicate that drought enhances iron transport and metabolic functions in the rhizosphere microbiome, suggesting a shift in the competitive dynamics for iron between host plants and soil microorganisms (Xu et al., 2021). This competition may further restrict the iron pool available to symbiotic rhizobia, exacerbating the stress-induced decline in nitrogen-fixing capacity.

Salinity alkalinity stress introduces an additional layer of complexity to iron homeostasis by altering soil chemistry and plant physiological processes. Alkaline environments often induce plant iron deficiency by elevating soil pH and promoting the formation of insoluble iron precipitates, thereby reducing iron bioavailability (Gao et al., 2024). In rice, salinity-alkali stress has been shown to reduce leaf photosynthetic performance and limit yield gains through iron deficiency, a physiological consequence that is likely conserved across plant species, including legumes. In soybean, salt stress activates stress response pathways that may interfere with iron signaling. The overexpression of ferritin (GmFER1) enhances salt tolerance by sequestering excess iron and mitigating oxidative damage, highlighting the subtle balance between iron sufficiency for symbiosis and iron toxicity under stress (Zhang et al., 2025). Furthermore, heavy metal contamination is often inversely correlated with iron concentrations in legumes. Mutations in iron transporter genes, such as IRT1, confer varying degrees of metal tolerance, indicating overlapping regulatory networks between iron and other metals (Liu et al., 2024b). High temperatures increase metabolic iron demand by accelerating respiration rates and the turnover of iron-containing proteins, while simultaneously compromising iron uptake and transport mechanisms (Yousaf et al., 2022).

The molecular mechanisms governing iron perception and signaling, particularly the BRUTUS (BTS) E3 ligase and Nodulation Signaling Pathway 1 (NSP1) axis, are also susceptible to modulation by abiotic stresses. In soybean, GmBTSa and GmBTSb act as Fe dependent molecular switches that stabilize GmNSP1a, thereby promoting nodulation (Gautam and Geddes, 2025). However, stress-induced alterations in cellular iron status or oxidative stress may perturb this stabilizing mechanism. The GSK3 kinase-mediated stress signaling pathway negatively regulates symbiotic nitrogen fixation in legumes through the specific phosphorylation of the key transcription factor GmNSP1. This mechanism reveals a direct molecular crosstalk and integration between abiotic stress signaling pathways and the Fe-dependent nodulation developmental cascade (He et al., 2021). Brassinosteroid signaling, which is frequently altered under stress, inhibits GmNSP1/2 activity, further coupling hormonal stress responses with Fe-regulated nodulation (Chen et al., 2023). The integration of these signaling pathways ensures the suppression of nodulation under adverse conditions, preventing the establishment of inefficient symbioses that would otherwise deplete plant resources without providing adequate fixed nitrogen (Ito et al., 2024). The combined effects of drought and heat stress induce significant alterations in antioxidant status and physicochemical properties, thereby disrupting the microaerobic environment required for nitrogenase activity. Since leghemoglobin synthesis is iron dependent, any stress induced reduction in iron availability compromises the oxygen-buffering capacity of root nodules, ultimately leading to the oxidative damage of nitrogenase (Sankari et al., 2022; Singh and Valdés-López, 2023). Therefore, the efficiency of symbiotic nitrogen fixation under heat stress is closely linked to the plant’s capacity to maintain iron homeostasis and protect iron containing proteins from oxidative degradation. Strategic optimization of iron bioavailability offers a promising avenue for enhancing the stress resilience of legume crops. Unlike indiscriminate iron supplementation, targeted approaches, such as the application of iron chelates, pH modulation can improve iron uptake and utilization efficiency under stress conditions (Wang et al., 2022). Besides soybean, other leguminous plants also exhibit similar iron-dependent stress response mechanisms. In Medicago truncatula, nodule-specific cysteine-rich (NCR) peptides not only guide bacteroid differentiation but also promote iron uptake by rhizobia through heme chelation. This mechanism may be impaired under stress conditions due to altered peptide expression levels (Singh and Valdés-López, 2023). In *Lotus japonicus*, IMA peptides regulate nodule formation and provide iron in response to internal nitrogen status, suggesting that the iron supply and nitrogen feedback loop constitute a conserved stress response module across multiple legume species (Ito et al., 2024). Targeting these mechanisms, the application of iron-based nanomaterials (e.g., Fe_3_O_4_) via foliar spraying or root treatment has been proven to promote nodule formation in soybean and common bean, improve yield and quality, and even enhance iron uptake and translocation under stress conditions (Wang et al., 2022). Furthermore, selecting cultivars with robust GmYSL7-mediated iron transport or elevated ferritin expression can enhance tolerance to both iron deficiency and abiotic stresses (Wu et al., 2023). Integrating iron management with stress alleviation strategies can sustain high levels of symbiotic nitrogen fixation and soybean productivity under increasingly severe environmental challenges. This approach reduces reliance on synthetic nitrogen fertilizers and promotes sustainable agriculture.

## 3 The absorption and transport mechanisms of iron in plants

### 3.1 Root absorption

Soil represents a vast reservoir of Fe, predominantly present as sparingly soluble Fe^3+^ oxides and hydroxides, particularly in calcareous and alkaline soils, where high pH and carbonate buffering severely restrict Fe bioavailability (Suzuki et al., 2021; Liang, 2022). Consequently, the concentration of plant available Fe^2+^/Fe^3+^ in the rhizosphere often falls below physiological thresholds required for essential processes including photosynthesis, respiration, nitrogen fixation, and antioxidant defense (Kabir et al., 2020; Murgia et al., 2022). To overcome this constraint, plants have evolved phylogenetically divergent, yet functionally convergent, Fe acquisition strategies. While the classical paradigm distinguishes Strategy I (non-graminaceous dicots and monocots) and Strategy II (graminaceous monocots), contemporary research reveals that Fe uptake mechanisms are farmore nuanced:multiple strategies coexist within species (Tsai and Schmidt, 2017);regulatory crosstalk integrates Fe signaling with other nutrient pathways (e.g., phosphate and zinc homeostasis); and root-microbiome interactions, including siderophore producing bacteria and mycorrhizal fungi, significantly augment Fe solubilization and mobilization. Thus, plant Fe nutrition is bet understood not as a binary classification but as a dynamic, context dependent integration of intrinsic genetic programs and extrinsic biogeochemical factors (Van Dijck et al., 2025).

**Figure 2:**
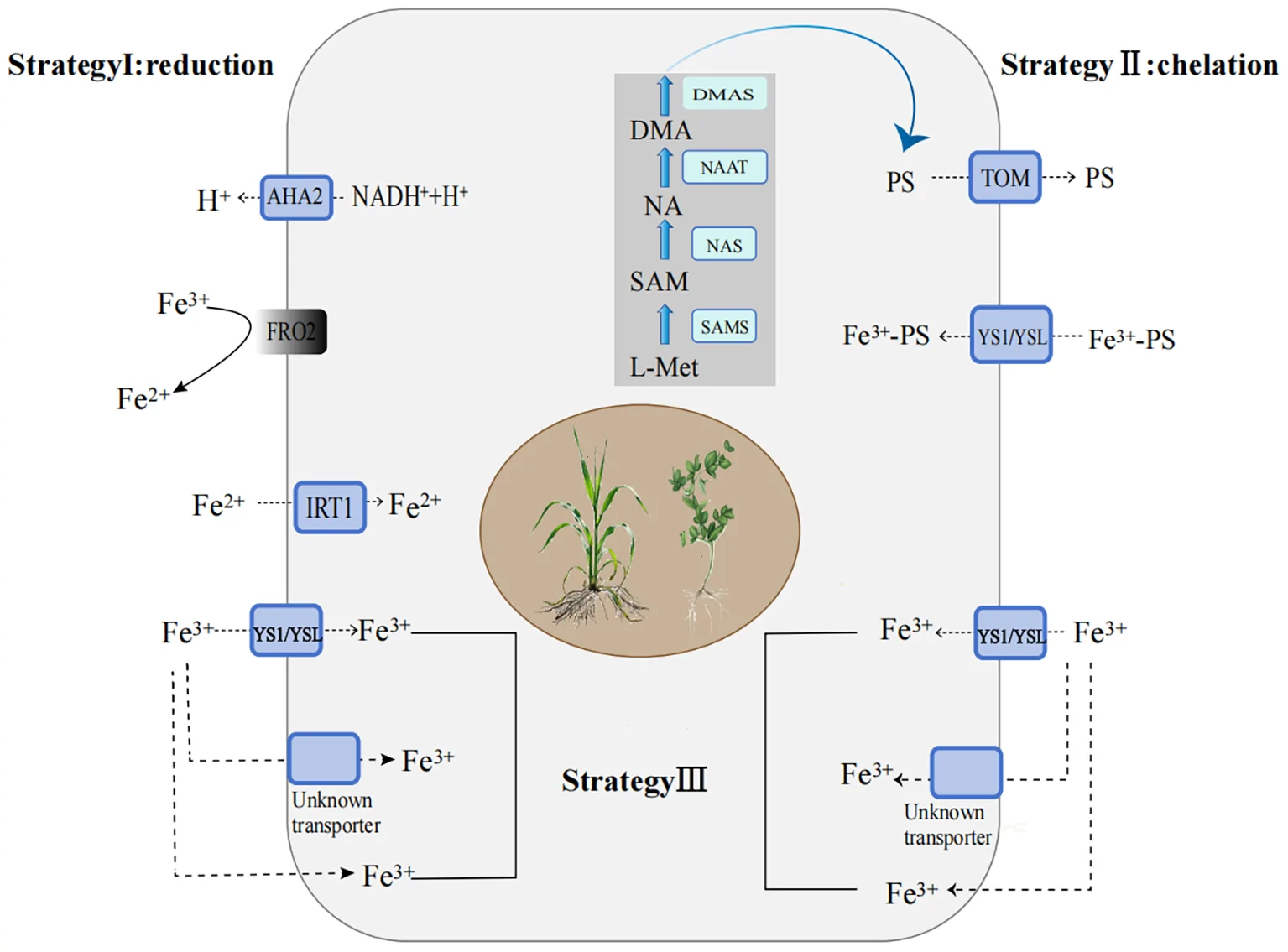
Schematic diagram of plant iron absorption and transport mechanisms. Strategy I (non - grass species): AHA2, Plasma Membrane H^+^-ATPase 2; FRO2, Ferric Reductase Oxidase; IRT1, iron-regulated transporters; Strategy II (grass species): L-Met, L-Methionine; SAM, S-adenosylmethionine; NA, nicotianamine; DMA, 2’-deoxymugineic acid; TOM, transporter of mugineic acid family phytosiderophores; PS, phytosiderophores; YS1/YSL, yellow stripe-like; Strategy III is a mixed type that integrates the core pathways of both mechanisms: 1) Direct absorption using plant iron carrier YS1/YSL transporters; 2) Absorption through unknown specific membrane transport proteins; 3) Absorption through vesicle - mediated endocytosis.

Plants employ three principal, mechanistically distinct strategies for acquiring Fe from soil (**Figure 2**), each reflecting evolutionary adaptation to specific edaphic constraints:

Strategy I (Reduction based uptake): Predominant in dicots and non-graminaceous monocots (including *Arabidopsis thaliana*), this pathway is activated under Fe deficiency. Root plasma membrane H^+^-ATPases acidify the rhizosphere, solubilizing sparingly available Fe^3+^ oxides/hydroxides (Oh et al., 2016). Subsequently, ferric chelate reductases, primarily FRO2, reduce Fe^3+^ to Fe^2+^ at the root surface; the resulting Fe^2+^ is then imported via the high affinity Fe^2+^ transporter IRT1 (Connolly et al., 2003). Both proton extrusion and Fe^2+^ uptake are energy dependent processes, ensuring efficient Fe acquisition under low bioavailability conditions. Notably, *Arabidopsis* also secretes coumarins (e.g., scopoletin and fraxetin), which form soluble Fe^3+^-coumarin complexes that resist precipitation and can be directly internalized, functionally extending Strategy I beyond canonical reduction (Van Dijck et al., 2025). Moreover, interspecific facilitation occurs: peanut (*Arachis hypogaea*) roots express AhYSL1, enabling uptake of maize secreted phytosiderophore-Fe^3+^ complexes, a clear demonstration of strategy hybridization in intercropping systems.

Strategy II (Chelation based uptake): Characteristic of graminaceous monocots (e.g., wheat, barley), this pathway relies on the synthesis, secretion, and reuptake of mugineic acid-family MAs (Gupta et al., 2021). Under Fe deficiency, MAs are synthesized in roots and secreted into the rhizosphere via TOM1 transporters. These high affinity Fe^3+^ chelators dramatically enhance Fe solubility in alkaline soils, and the resulting MA-Fe^3+^ complexes are imported exclusively through YS1/YSL family transporters (Qi et al., 2024). Rice (*Oryza sativa*) exhibits remarkable plasticity: while it expresses YS1 and utilizes MAs under aerobic conditions, it additionally deploys OsIRT1 to directly absorb Fe^2+^ under flooded, anaerobic conditions, thereby integrating Strategy I functionality into its otherwise Strategy II dominant framework (Kobayashi and Nishizawa, 2012; Judesse Soviguidi et al., 2025).

Strategy III (Microbial siderophore mediated uptake): Emerging evidence identifies a third, ecologically significant pathway: direct acquisition of microbially derived siderophores (Gu et al., 2025). Soil bacteria, including *Bacillus* and *Pseudomonas* spp, secrete hydroxamate or catecholate type siderophores that chelate Fe^3+^ with extraordinary affinity. Plants may internalize these Fe siderophore complexes via (i) co-option of endogenous YS1/YSL transporters, (ii) unidentified high affinity membrane carriers, or (iii) clathrin mediated endocytosis. Critically, rhizospheric siderophore concentrations often exceed plant Fe demand, positioning the root microbiome as a functional extension of the plant’s Fe acquisition apparatus. Collectively, these strategies underscore that plant Fe nutrition is not governed by rigid taxonomic rules but by dynamic, context responsive integration of intrinsic genetic programs and extrinsic biogeochemical-microbial interactions. The absorption and transport of iron by plants are controlled by a complex molecular regulatory network (Rai et al., 2021). Under iron deficiency conditions, plants will upregulate the expression of related genes such as *FRO2, IRT1, and NAS* (nicotinamide synthase), and plant hormones such as ethylene and jasmonic acid will also regulate the expression of iron absorption genes by activating transcription factors (Cui et al., 2018; Nanda et al., 2024). After iron is absorbed into root cells, it is transported to the aboveground parts through the xylem and is preferentially distributed to areas with high demand, such as new leaves and growth points. Excess iron is stored in ferritin. In addition, the absorption of iron by the root system is also influenced by environmental factors such as soil pH value, temperature, aeration conditions, and the interaction between elements. Reasonable soil management and fertilization strategies are crucial for improving the efficiency of iron absorption.

### 3.2 Distribution within the body

After root uptake, iron is systemically distributed throughout plants via the vascular system, with xylem primarily responsible for long-distance transport to shoot tissues and phloem mediating iron redistribution to sink organs. In the xylem, loaded Fe^2+^ must be oxidized to Fe^3+^ to form stable chelates with citrate, ensuring solubility and transport efficiency. Multicopper oxidases LPR1 and LPR2, localized at xylem vessel walls, catalyze the oxidation of Fe^2+^ to Fe^3+^. This process not only maintains the stability of Fe^3+^-citrate complexes in the xylem sap but also prevents Fe^2+^-mediated Fenton reactions, thereby enabling efficient long-distance iron translocation (Xu et al., 2022). Loss of LPR1 and LPR2 function leads to elevated Fe^2+^ levels in xylem sap, resulting in aberrant iron deposition on vessel walls and severely impairing iron allocation to developing leaves (Zhu et al., 2022). In the phloem, iron is predominantly transported as Fe^2+^-NA complexes, with YSL family transporters mediating phloem loading and unloading to direct iron delivery to low-transpiration sink tissues, including young leaves, floral organs, and seeds (Zulfiqar et al., 2023).

Notably, stress conditions profoundly alter vascular iron transport and allocation patterns. Under drought stress, diminished transpiration reduces xylem driving force, leading to decreased iron translocation to aerial parts. Drought also induces structural modifications in xylem and phloem tissues; treatment with iron oxide nanoparticles prevents drought-induced epidermal and xylem shrinkage, maintaining the patency of metaxylem and phloem, thereby facilitating nutrient distribution under stress conditions (Ndou et al., 2023). Studies in sorghum have revealed that drought stress can significantly increase seed iron content and upregulate expression of vacuolar iron transporters and ferritin genes, suggesting a reprogramming of iron redistribution strategies under water deficit. Similarly, salt stress imposes osmotic constraints that adversely affect iron uptake and translocation, with plants dynamically regulating acquisition and homeostasis through transcription factors such as FIT to accommodate nutritional demands (Araki et al., 2022). Nutrient stress also disrupts phloem function; when ammonium is supplied as the sole nitrogen source, LPR2-dependent iron over-accumulation in the phloem apoplast triggers reactive oxygen species bursts and callose deposition, inhibiting primary root growth (Liu et al., 2022). This reveals a pathological mechanism of phloem iron redistribution under nutrient stress. Recent studies have further demonstrated that plants employ long-distance signaling via the vascular system to coordinate systemic responses under mineral nutrient stress; cytokinin are loaded into xylem through ABCG14 for shootward signaling, while phloem-mobile small RNAs, such as miR399 in phosphate deficiency, serve as systemic signals (Buhtz et al., 2010; Xia et al., 2025). Under iron deficiency, silicon promotes phloem remobilization of iron from old to young leaves by enhancing nicotianamine synthesis and YSL1 expression (Pavlovic et al., 2016), while abscisic acid (ABA) alleviates iron deficiency symptoms through promoting root development and reallocating iron to shoots (Zhang et al., 2020a). Citrate and NA act synergistically in xylem-phloem iron translocation; studies in the Arabidopsis frd3 zif1 double mutant demonstrate that simultaneous disruption of citrate xylem loading and NA subcellular compartmentalization results in severe Fe-Zn homeostasis imbalance, root architecture defects, and oxidative stress. Collectively, vascular iron transport under stress is not merely a passive process subject to physical hindrance, but rather a dynamically reprogrammed process involving multi-layered active regulation. Drought affects xylem transport efficiency by reducing transpirational driving force and altering vascular architecture, while potentially activating phloem-mediated remobilization to secure iron supply to sink organs. Conversely, salt and nutrient stress modify iron partitioning between xylem and phloem through ionic competition, redox imbalance, and signaling interference. To cope with these challenges, plants execute adaptive modulation via transcriptional regulatory networks, hormonal signals, and small RNAs.

### 3.3 Subcellular localization

Fe homeostasis in plants requires not only inter-organ distribution but also precise subcellular compartmentalization. To prevent Fe-catalyzed oxidative damage, particularly via Fenton chemistry, excess cytosolic Fe is sequestered into the vacuole, a major intracellular Fe storage organelle (Thomine and Vert, 2013; Sharma et al., 2016). This sequestration is mediated by tonoplast-localized transporters: VIT1 facilitates Fe^2+^ import into the vacuole and determines Fe distribution patterns in seeds (Ram et al., 2021); MTP8 a member of the Mn-CDF family localized to the tonoplast, contributes to vacuolar Fe^2+^/Mn^2+^ compartmentalization (Chu et al., 2017); NRAMP3 and NRAMP4, also tonoplast localized, mediate Fe remobilization from the vacuole during germination and Fe deficiency responses (Cvitanich et al., 2010). Collectively, these transporters maintain organelle-specific Fe pools and safeguard cellular redox integrity during abiotic stress (Zhu et al., 2016).

Within the vacuole, Fe is stored in dynamic redox states, often complexed with organic acids (e.g., citrate, malate) or phytate, forming bioavailable yet nontoxic chelates (Ibeas et al., 2017; Hoang et al., 2021). Separately, and in a spatially distinct pathway, Fe is stored within plastids in the protein nanocage of ferritin, a 24-subunit spherical complex capable of storing up to 4,500 Fe atoms as ferrihydrite (Grillet et al., 2014). Plant ferritins are characterized by their plastid specific cellular localization and transcriptional regulation by iron (Briat et al., 1999). Recent work has confirmed that the full-length rice ferritin OsFER2 and its transit peptide are localized to the chloroplast (Nguyen et al., 2022), and ferritins function as critical iron buffering proteins within this organelle, playing an important role in shaping the chloroplast ionome (Holzner et al., 2026). In Arabidopsis senescing leaves, ferritin mediates transient chloroplast iron sequestration to enable iron recycling (Gracheva et al., 2026), further reinforcing that ferritin functions primarily within the plastid compartment. Thus, vacuolar Fe sequestration (mediated by VIT1, MTP8, and organic acid chelation) and ferritin- mediated Fe storage (confined to plastids) represent two mechanistically and spatially distinct iron buffering systems (Cao, 2019).

Upon demand, vacuolar Fe is mobilized primarily via NRAMP3 and NRAMP4 transporters, as well as reductase-coupled mechanisms involving the tonoplast transporter AtDTX25, releasing Fe^2+^ into the cytosol. The remobilization of vacuolar Fe stores is essential during germination and early seedling establishment (Murgia et al., 2022). Subsequently, Fe^2+^ is delivered by chaperones such as NA and transporters including PIC1 (plastid iron carrier, localized to the plastid inner envelope) to Fe-avid compartments like chloroplasts and developing pollen. Critically, spatial regulation of transporter expression, both tissue-specific (e.g., elevated VIT1/VIT2 in roots and seeds, ferritin in photosynthetically active leaves) and subcellular (tonoplast localized VIT1/NRAMP vs. plastid envelope-localized PIC1 and plastid targeted ferritin), ensures preferential Fe allocation to metabolically active sinks (Aung and Masuda, 2020), thereby sustaining energy metabolism, reproductive development, and stress resilience.

## 4 The toxic effects of iron ions under abiotic stress

### 4.1 Oxidative damage caused by excessive iron

Abiotic stressors such as soil waterlogging, low pH, elevated temperature, and industrial contamination can dramatically increase rhizospheric concentrations of soluble Fe^2+^, converting iron from an essential micronutrient into a potent cytotoxic agent. Iron toxicity is a widespread mineral disorder particularly prevalent in anaerobic soils (Wu et al., 2019). Upon excessive uptake, labile Fe^2+^ participates in Fenton reactions to generate hydroxyl radicals (•OH) and other ROS, which exert indiscriminate oxidative damage to proteins, membrane lipids, and DNA, ultimately leading to ROS-mediated cell death and disruption of photosynthetic apparatus (Zahra et al., 2021).

Soil pH is the single most influential edaphic factor governing Fe^2+^ availability and toxicity severity. Fe toxicity often occurs in rice grown in submerged paddy fields with low pH, leading to dramatic increases in ferrous ion concentration, disrupting cell homeostasis and impairing growth and yield. The pH-dependent solubility of iron follows a well-defined pattern: under acidic conditions, Fe^3+^ reduction to Fe^2+^ is thermodynamically favored, and the solubility of Fe(OH)_3_ increases by approximately 1,000-fold per unit decrease in pH (Moraghan and Mascagni, 1991). For instance, experimental evidence from graphene oxide-treated rice demonstrates that solution acidification serves as the main driver for Fe overload in plants, GO-induced acidification of the nutrient solution caused excessive Fe accumulation in shoots (2.2 and 3.6 times higher than control at 100 and 250 mg/L GO, respectively), which was found to be the main reason for the oxidative damage in shoots (Zhang et al., 2020a). This creates a feed-forward amplification loop in acid soils where low pH simultaneously increases Fe^2+^ supply and accelerates its conversion to cytotoxic radicals. Iron toxicity is more likely to occur in soils with high organic matter or elevated levels of available iron, conditions frequently co-occurring with acidic pH (Zhang et al., 2020b). Flooding or waterlogging creates anaerobic conditions that fundamentally shift soil redox chemistry toward Fe^3+^ reduction, generating toxic Fe^2+^ concentrations that can exceed plant tolerance thresholds (Pinto et al., 2016). Under waterlogged conditions, soluble iron present in the soil solution is absorbed by roots and accumulates in leaves, causing poor growth and tillering and severe yield reductions.

In addition to pH and redox potential, soil temperature directly affects the rate of microbial Fe^3+^ reduction in waterlogged soils (Liu et al., 2020), with higher temperatures accelerating anaerobic microbial activity and consequently increasing Fe^2+^ production rates. Furthermore, elevated temperatures increase ROS generation rates independently of Fe status by accelerating metabolic flux through mitochondrial and chloroplast electron transport chains (Xie et al., 2019), thus narrowing the threshold between tolerable and toxic Fe concentrations. Plants exposed to combined heat and Fe excess face a dual challenge: heightened ROS production from thermal stress combined with enhanced Fenton reactivity from increased Fe availability.

The convergence of these environmental factors determines the severity of Fe toxicity manifestation. Fe toxicity causes severe morphological and physiological disorders, including reduced germination percentage, interference with enzymatic activities, nutritional imbalance, membrane damage, and chloroplast ultrastructure disruption (Zahra et al., 2021). These findings establish that Fe toxicity in plants cannot be understood solely through the lens of cellular Fenton chemistry; rather, it is an environmentally contingent phenomenon whose severity is governed by the interaction between soil physicochemistry (pH, Eh, temperature) and plant genotype-specific tolerance mechanisms. Mitigation strategies must therefore target multiple levels: (i) In waterlogged lowland and acid sulfate soils (pH < 4.5), high Fe^2+^ bioavailability often coincides with Al^3+^ toxicity. Integrated management strategies include intermittent drainage or alternate wetting-and-drying (AWD) to promote rhizosphere aeration, thereby oxidizing soluble Fe^2+^ into insoluble Fe^3+^ precipitates; in acidic profiles, organic amendments (e.g., straw or Eleocharis dulcis compost) are more effective than dolomite alone in buffering pH and reducing Fe^2+^ activity, while liming should be tailored to soil buffering capacity and crop-specific requirements to avoid secondary nutrient disorders. Potassium fertilization can partially restore K/Fe homeostasis and limit root Fe^2+^ uptake; (ii) For long-term resilience, genetic improvement remains the most sustainable approach, relying on cultivars with enhanced root oxidizing power, elevated vacuolar Fe sequestration via upregulated VIT1 expression, or superior antioxidant capacity (tissue-tolerance). Under acute Fe stress, exogenous application of ascorbate, α-tocopherol, or brassinosteroids (e.g., EBR) can transiently restore redox balance, with EBR increasing root aerenchyma area by approximately 70% under toxic Fe levels, thus facilitating internal O_2_ transport and Fe^2+^ oxidation at root surfaces. Endogenously, excess Fe strongly induces OsNAS3 overexpression, and the resulting NA chelation serves as a crucial internal detoxification pathway.

### 4.2 Iron deficiency and growth inhibition

**Figure 3:**
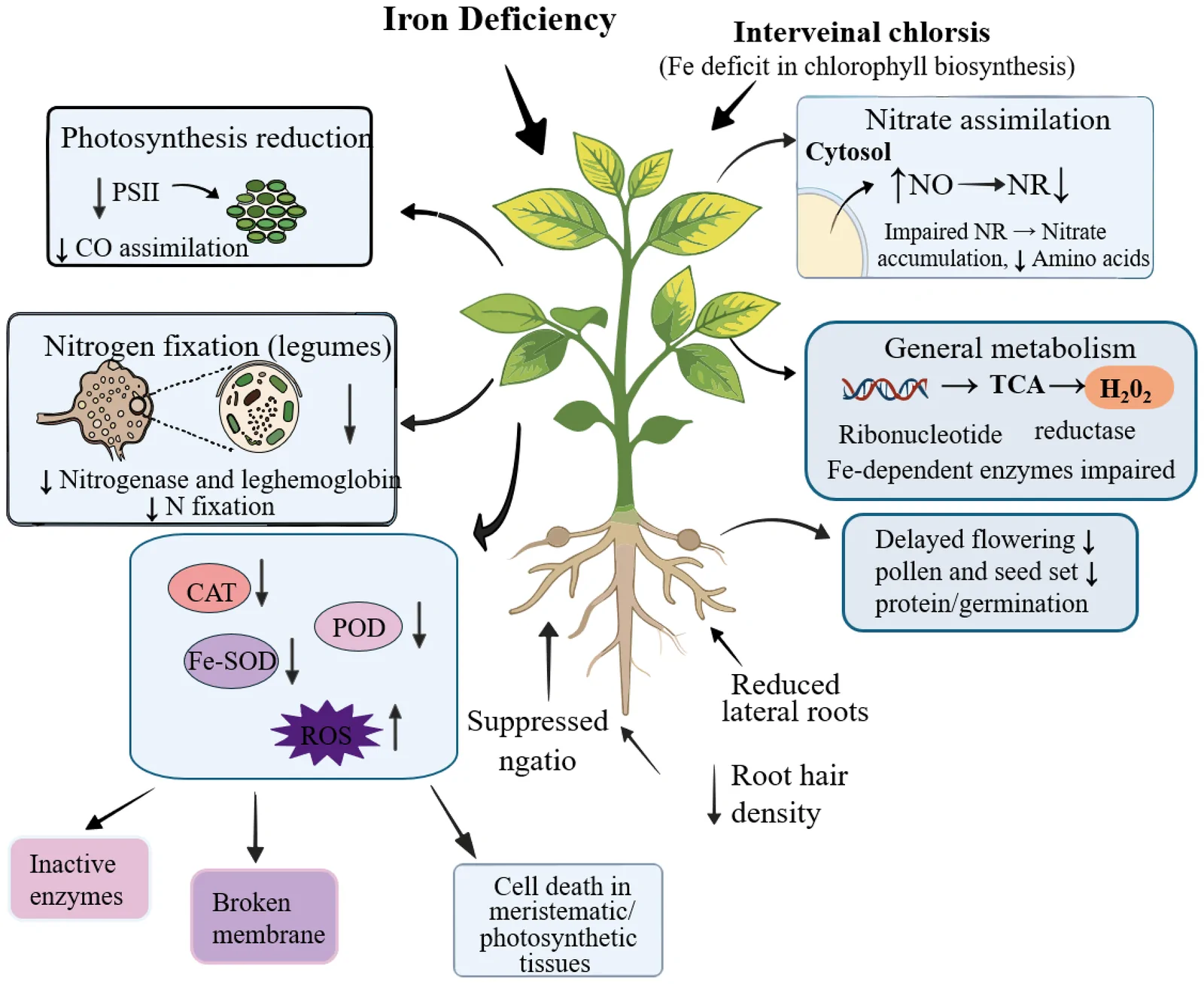
Schematic diagram summarizing the physiological and metabolic alterations in plants under iron deficiency stress. NR, nitrate reductase; Fe-SOD, iron-dependent superoxide dismutase; CAT, catalase; POD, peroxidase; ROS, reactive oxygen species; TCA, tricarboxylic acid cycle; PSII, photosystem II.

Under abiotic stress, particularly in calcareous, alkaline rhizospheric Fe bioavailability is severely constrained by elevated pH (which promotes Fe^3+^ hydroxide precipitation) and altered redox potential (e.g., reductive conditions that paradoxically limit Fe^2+^ oxidation necessary for Strategy I uptake) (Molnár et al., 2023; Li et al., 2024a). Iron deficiency thus manifests as a syndrome of physiological and developmental impairments (**Figure 3**). The most prominent feature is interveinal chlorosis in young leaves, compromises the integrity of photosystem II (PSII), thereby impairing photosynthesis and carbon fixation. Meanwhile, as an essential component of nitrate reductase and nitrogenase, severely restricts nitrogen assimilation and symbiotic nitrogen fixation efficiency (Gao et al., 2022; Therby-Vale et al., 2022; Tran et al., 2023; Zhou et al., 2024). Beyond these core pathways, Fe serves as an essential cofactor for over 300 enzymes, including ribonucleotide reductase (DNA synthesis), aconitase (TCA cycle), and peroxidases, whose functional impairment disrupts central metabolism, cell division, and redox signaling (Denic et al., 2023). Root architecture is profoundly affected: primary root elongation is suppressed, lateral root initiation and emergence are diminished, and root hair density declines, which collectively reduces hydraulic conductivity and nutrient foraging capacity (Lešková et al., 2022). Reproductive development is equally vulnerable: Fe deficiency delays floral transition, reduces pollen viability and stigma receptivity, lowers seed set percentage, and compromises seed protein content and germination vigor (Chen et al., 2021). Critically, Fe is integral to the catalytic centers of key antioxidant enzymes, including CAT, POD, and Fe-SOD, and its deficiency therefore undermines cellular ROS scavenging capacity, exacerbating oxidative damage and destabilizing redox homeostasis (Xu et al., 2025).

Fe deficiency compromises the catalytic integrity of Fe-dependent antioxidant enzymes (CAT, POD, and Fe-SOD), thereby diminishing cellular ROS scavenging capacity. This results in progressive accumulation of O_2_^•^-^^, H_2_O_2_, and ^•^OH, culminating in oxidative stress that disrupts membrane integrity, inactivates metabolic enzymes, arrests cell cycle progression, under chronic or severe deficiency, induces programmed cell death in meristematic and photosynthetic tissues (Kroh and Pilon, 2020). In response, plants deploy multilayered adaptive strategies: (i) rhizosphere acidification via plasma membrane H^+^-ATPase activation to solubilize Fe^3+^ oxides; (ii) enhanced secretion of Fe chelating compounds; and (iii) transcriptional upregulation of Fe transporters, including *IRT1, FRO2, YSLs*, and *VITs*, to augment uptake, reduction, chelation, and subcellular compartmentalization (Rodrigues et al., 2023). Nevertheless, these endogenous mechanisms are frequently insufficient under intense abiotic stress (e.g., pH > 7.5, high bicarbonate, or prolonged drought), necessitating targeted anthropogenic interventions. Agronomic approaches include: (i) soil amelioration, such as application of elemental sulfur or ammonium based fertilizers to lower rhizosphere pH, or incorporation of composted organic matter to improve cation exchange capacity and Fe chelating ligand supply; and (ii) direct foliar supplementation with bioavailable Fe formulations, e.g., Fe-EDDHA (ethylenediamine-N,N′-bis (2-hydroxyphenylacetic acid) or Fe-NA (nicotianamine) complexes, which bypass root uptake limitations and rapidly restore Fe status in young leaves and reproductive organs. Such integrated management, leveraging both plant physiological plasticity and precision agronomy, represents the most effective pathway to sustain productivity under Fe limiting conditions.

### 4.3 The harmful effects of iron homeostasis imbalance

Abiotic stress profoundly disrupts plant iron (Fe) homeostasis, impacting Fe acquisition, long distance translocation, subcellular compartmentalization, and redox regulation in an integrated, system wide manner (Gupta et al., 2026). Under optimal conditions, Fe homeostasis is maintained through a tightly coordinated transcriptional–posttranslational regulatory network: key genes, including *IRT1, FRO2, NAS, YSLs*, and *VIT1* are dynamically regulated by Fe sensing transcription factors (e.g., FIT, bHLH Ib proteins, and BTS-family E3 ligases) to ensure balanced Fe flux across root-shoot-organelle interfaces (Hodgens et al., 2021). This regulatory architecture enables efficient Fe utilization, recycling, and storage (primarily as ferritin nanocages in vacuoles or plastids), while minimizing oxidative risk. However, abiotic stressors, including drought, salinity, extreme temperatures, and heavy metal contamination, compromise this equilibrium via multiple non-exclusive mechanisms: (i) drought induced stomatal closure and root hydraulic limitation reduce rhizosphere exploration and Fe^2+^/Fe^3+^ solubilization; (ii) salinity elevates cytosolic Na^+^ and Cl^-^, disrupting membrane potential and impairing H^+^-ATPase driven proton extrusion essential for Strategy I Fe uptake; (iii) heat stress denatures Fe binding proteins (e.g., ferredoxins) and destabilizes Fe-S clusters, increasing labile Fe pools and ROS generation; and (iv) oxidative stress itself feeds back to inhibit Fe transporter expression and promote Fe mislocalization (Wang et al., 2014; Nazari et al., 2023). Consequently, Fe distribution becomes spatially dysregulated, characterized by Fe hyperaccumulation in older tissues (e.g., mature leaves, root vacuoles) and Fe deficiency in metabolically active sinks (e.g., meristems, young leaves, developing seeds), thereby amplifying cellular damage (Shi et al., 2022). Critically, Fe dyshomeostasis does not occur in isolation: it synergizes with other nutrient imbalances, such as Zn^2+^ and Mn^2+^ competition for IRT1-mediated uptake, or P-Fe antagonism in alkaline soils to generate compound stress phenotypes (Martín-Barranco et al., 2020; Spielmann and Vert, 2021). Moreover, Fe deficiency impairs the function of Fe dependent enzymes involved in N, S, and C metabolism (e.g., nitrate reductase, sulfite reductase, aconitase), thereby propagating systemic nutritional imbalance (Vigani and Murgia, 2018; Liu and Xu, 2023). Thus, Fe homeostasis functions as a central node within the plant ionome, a dynamic, interdependent network whose integrity is indispensable for stress resilience and productivity.

## 5 Conclusions

Fe serves not only as an essential micronutrient but also as a central metabolic cofactor, redox regulator, and signaling hub, integrally coordinating photosynthesis, respiration, antioxidant defense, and legume, rhizobia symbiosis under abiotic stress. This review synthesizes emerging evidence demonstrating that Fe homeostasis functions as a core regulatory node within the plant ionome network, dynamically orchestrated by Fe-sensing transcription factors (FIT, bHLH Ib, BTS), post-translational stabilization of key regulator, including GmNSP1a and extensive crosstalk with hormonal and nutrient signaling pathways. Plant Fe acquisition encompasses Strategy I (reduction-based), Strategy II (chelation-based), and microbiome-assisted Strategy III, collectively reflecting an evolutionary plastic, context-dependent nutritional system shaped by genotype-soil-microbiota interactions. Long-distance Fe transport relies on xylem-localized citrate Fe³^+^ complexes and phloem-localized nicotiana mine Fe^2+^ complexes, while subcellular sequestration into ferritin nanocages and vacuolar compartments critically balances Fe bioavailability against Fenton reaction-mediated oxidative toxicity. Abiotic stresses perturb this finely tuned equilibrium, leading to spatially heterogeneous Fe distribution and exacerbated oxidative damage; conversely, optimal Fe status bolsters stress resilience by reinforcing coordinated antioxidant responses and potentiating stress-responsive signaling cascades. Future research priorities include resolving Fe-sensing mechanisms under combined abiotic stresses, defining mechanistic bases of Fe-microbe-plant tripartite interactions, establishing causal links between Fe homeostasis genes and quantifiable stress tolerance phenotypes, and translating these insights into precision agronomic strategies for climate-resilient crop production.
