# Supplementary material for: Iron need attention: a review on the role of iron in plants
Source: Front Plant Sci. 2026 Sep 11;17:1876801. doi: 10.3389/fpls.2026.1876801 (PMC13613640; doi:10.3389/fpls.2026.1876801)
Supplement: Supplementary file 1 [file Table1.docx]

**Supplementary Table S1.**

*Summary of iron-related genes and their regulatory mechanisms in plants.*

| Gene/  Protein | Species | Biological Function | Pathway/Regulatory Mechanism | References |
| --- | --- | --- | --- | --- |
| *FIT* | *Arabidopsis* | Central regulator of Fe deficiency; activates *IRT1* and *FRO2* transcription | bHLH network; ROS negative feedback | (Von Der Mark et al., 2021) |
| *IRT1* | *Arabidopsis* | Plasma membrane divalent Fe transporter; root Fe uptake | FIT target; inhibited by CARK3/bHLH106 phosphorylation | (Yu et al., 2026) |
| *FRO2* | *Arabidopsis* | Plasma membrane ferric reductase; reduces Fe^3+^ to Fe^2+^ | FIT target; core component of Strategy I | (Riaz and Guerinot, 2021) |
| *bHLH106* | *Arabidopsis* | Transcription factor; inhibits *IRT1* upon phosphorylation | Post-translational modification; prevents Fe overload | (Yu et al., 2026) |
| *StbHLH47* | Potato | Regulates Fe-related genes; enhances tuber Fe content when edited | Conserved bHLH function; biofortification target | (Chauhan et al., 2024) |
| *GhbHLH121* | Cotton | Iron deficiency response network hub gene | Induces expression of multiple downstream iron absorption genes | (Li et al., 2023) |
| *YSL15* | Rice/Wheat | Iron-deoxymugineic acid carrier transporter protein, mediates iron uptake | Strategy II iron absorption | (Li et al., 2023) |
| *NAS2* | Wheat | Nicotianamine synthase, synthesizes iron chelators | Pyramiding with YSL15 and other genes to increase grain iron and zinc content | (Gao and Dubos, 2021) |
| *RTH* | *Arabidopsis* | Activates iron assimilation-related genes, promotes absorption and transport | Possible crosstalk with ethylene signaling pathway | (Qi et al., 2024) |
| *MbYSL7* | Apple | Mediates Fe transport; interacts with *MbHY5* for chlorophyll synthesis | Light signaling and Fe homeostasis crosstalk | (Sun et al., 2022) |
| *GLU1* | *Arabidopsis* | Glutamate synthase, participates in iron deficiency response and long-distance transport | Intersection of nitrogen metabolism and iron homeostasis; mutation leads to abnormal iron distribution | (Cui et al., 2020) |
| *MIT1/2* | *Arabidopsis* | Mitochondrial iron transporter protein | Mitochondrial iron homeostasis; deletion leads to severe growth defects | (Vargas et al., 2023) |
| *COS* | *Arabidopsis* | Long non-coding RNA, expression induced by iron deficiency | Post-transcriptional regulation; affects systemiciron homeostasis levels | (Bakirbas and Walker, 2022) |
| *MYB308* | Citrus | Activates plasma membrane A^+^-ATPase 6, promotes proton extrusion | Rhizosphere acidification mechanism; enhances iron availability | [(Fan et al., 2022)](http://dx.doi.org/10.1093/HR/UHAC088) |
| *SOD7/DPA4* | *Arabidopsis* | Transcriptional repressor, restricts organ growth to coordinate iron uptake | SOD7/DPA4-GIF1 module; balances growth and nutrition | (Zheng et al., 2023) |
| *AtHAP5A* | *Arabidopsis* | HAP transcription factor, regulates iron transport from root to shoot | Iron transport regulatory network | (Zheng et al., 2023) |
| *CRR* | *Arabidopsis* | CYBDOM protein, promotes ascorbate-dependent ferric reduction | Regulation of ferric iron deposition at root tips under phosphate deficiency | (Clúa et al., 2024) |

**Note.** Gene names are italicized. The table summarizes core components including transcription factors (e.g., *FIT*, *bHLH*), transporters (e.g., *IRT1*, *YSL15*), and enzymes (e.g., *FRO2*, *NAS2*) involved in iron uptake, translocation, and signaling. Strategy I refers to the reduction-based mechanism in dicots, while Strategy II refers to the phytosiderophore-based mechanism in graminaceous plants.

**Source:** Compiled from relevant literature.
